# Clinical, Pathological, and Prognostic Features of Male Breast Cancer: A Multicenter Study

**DOI:** 10.3390/curroncol30110716

**Published:** 2023-11-11

**Authors:** Francesca Accomasso, Silvia Actis, Carola Minella, Roberta Rosso, Claudia Granaglia, Riccardo Ponzone, Nicoletta Biglia, Valentina Elisabetta Bounous

**Affiliations:** 1 Gynecology and Obstetrics Unit, Mauriziano Umberto I Hospital, Department of Surgical Sciences, University of Turin, 10128 Turin, Italy; francesca.accomasso@unito.it (F.A.); silvia.actis@unito.it (S.A.); carola.minella@unito.it (C.M.); roberta.rosso410@edu.unito.it (R.R.); valentinaelisabetta.bounous@unito.it (V.E.B.); 2 Department of Surgical Sciences, University of Turin, 10124 Torino, Italy; 3 Candiolo Cancer Institute, FPO-IRCCS, 10060 Candiolo, Italy; riccardo.ponzone@ircc.it

**Keywords:** male breast cancer, breast cancer surgery, adjuvant endocrine therapy, breast cancer survival

## Abstract

Male breast cancer (BC) represents less than 1% of male tumors. Little is known about male BC characteristics, management, and survival, with many studies based on a small number of cases. Consequently, the treatment of male BC lacks specific guidelines. The aims of the study are to compare male and female breast cancer (FBC) in terms of cancer clinical and anatomopathological features and treatment approach, and to identify differences between male BC and FBC in terms of survival. Patients and methods: Data from 2006 to 2018 were retrospectively acquired. Amounts of 49 males and 680 postmenopausal females with primary non-metastatic BC who underwent breast surgery at Mauriziano Hospital or IRCCS Candiolo (TO—Italy) were included. The mean age at diagnosis for male BC was 68.6 years, and males presented a smaller tumor size than women (*p* < 0.05) at diagnosis. Most male BC patients received adjuvant endocrine therapy (AET) with tamoxifen (73.5%). AET drop-out rate due to side effects was 16.3% for males compared to 7.6% for women (*p* = 0.04). Comparing FBC and male BC, no differences have been identified in terms of DFS and OS, with a similar 10-year-relapse rate (12% male BC vs. 12.4% FBC). Propensity Score Matching by age, nodal status, pT, and molecular subtype had been performed and no differences in OS and DFS were seen between male BC and FBC. In conclusion, male BC and FBC have similar prognostic factors and survival outcomes. The drop-out rate of AET was higher in males, and side effects were the main reason for drug discontinuation.

## 1. Introduction

Breast cancer (BC) is the most common malignant tumor among women worldwide; many researches have been conducted on this subject. Male breast cancer represents less than 1% of male tumors; in Italy, 500 new cases were recorded in 2018 [1]. Due to its rarity, there are limited information about male BC characteristics, management, and survival, with many studies based on a small number of cases. Consequently, the treatment of male BC lacks specific guidelines.

Males share with females the principal risk factors of developing BC such as age, smoking, and alcohol consumption; in addition, all the causes of hyperestrogenism as cirrhosis, obesity, testicular injuries, or Klinefelter syndrome can increase the risk of BC in males [2,3]. Inherited genetic mutations are a relevant risk factor for male BC; studies in the literature indicate that 15–20% of sick patients present hereditary breast or ovarian cancer, this suggests a significant role of genetic factors in predisposition for male BC [4,5]. About 10% of male BC patients have a genetic susceptibility, while, among women, hereditary tumors only represent 5–7% [6,7]. The most common predisposing genetic mutations for male BC are BRCA1 and BRCA2, CHECK2 and MLH1, and MSH2 and MSH6 (related to Lynch syndrome) [8]. In particular, BRCA2 seems to play a primary role as men with a BRCA2 mutation have a lifetime risk of developing breast cancer of about 5–10% [6,7].

Data in the literature are conflicting: in most studies, there are no differences between males and females in terms of prognosis [9]. Conversely, other studies showed a worse outcome for male BC [10,11]. Indeed data on survival are very heterogeneous through the literature since the survival rate of MBC varies between 36 and 66% and overall survival (OS) in male BC appears lower than in women (80–86%) [12].

Male BC usually presents as a unilateral breast lump and undergoes mastectomy in most cases [3].

Lacking specific treatment guidelines and indications for male BC, adjuvant treatments are the same as those used in female breast cancer (FBC). This practice may be suboptimal in some cases considering that hormonal differences between men and women may influence the effectiveness of treatments. For this reason, some aspects of male BC need improvement in specific management, such as surgery, radiotherapy, and systemic treatments [2,12].

This study aims to compare male and female BC in a cohort of real-life patients, analyzing clinical and anatomopathological features of the tumors and the treatment approach. The goal is to identify differences and possible implications to improve disease management and survival.

## 2. Materials and Methods

### 2.1. Study Population and Biomarkers Evaluation

We retrospectively compared 49 male patients to 680 female postmenopausal patients with a minimum follow-up available of 36 months with primary non-metastatic BC. All the patients underwent breast surgery at the Academic Department of Obstetrics and Gynaecology of “Umberto I” Hospital in Torino and IRCCS Hospital in Candiolo between 1 January 2006 and 31 December 2018.

Data regarding the patient’s medical history, age, ongoing therapies, previous surgery, personal and hereditary oncological history, BRCA1/2 gene mutation, clinical and biological characteristics of the tumor, and follow-up data were obtained from prospectively maintained, non-open-access institutional databases.

As recommended by the most recent version of the NCCN guideline, all the specimens had been tested for hormone receptors such as estrogen (ER) and progesterone receptors (PgR) by immunohistochemistry (IHC). Cancers were considered hormone-receptor-positive if at least 1% of the cells tested expressed ER. Otherwise, the tumor was considered hormone receptor-negative [13,14,15]. Human Epidermal Growth Factor Receptor 2 (HER2) overexpression was evaluated with IHC staining 3+. In the case of IHC equivocal results (2+), amplification in situ hybridization (FISH) test was performed [16].

We classified both male and female BC using IHC surrogates for molecular subtypes, as follows: luminal A (ER and/or PgR positive and HER-2 negative; ki67 < 20%), luminal B (ER and/or PgR positive, HER-2 positive and ki67 > 20%), HER-2 (ER and PgR negative and HER-2 positive) and basal-like (ER, PgR and HER-2 negative) [17,18].

Surgical and adjuvant medical treatment decisions were based on current international guidelines at the time of surgery [19,20,21]. Patients underwent clinical follow-up every 6 months in the first 5 years after surgery and once a year subsequently.

Consent to the anonymous use of their clinical and instrumental data for scientific purposes is routinely signed by all patients treated at both institutes.

### 2.2. Statistical Analysis

The Kolmorogov–Smirnov test was performed to evaluate the normal distribution of the variables. The association and the statistical significance of the categorical variables have been verified with Chi-square or Fisher’s exact test, when applicable. The t-Student test has been used to compare continuous variables with normal distribution.

The survival endpoints were Disease Free Survival (DFS) and Overall Survival (OS) and Kaplan–Meier curves and log-rank tests were carried out in the two groups. Univariate and multivariate Cox regression analyses were also performed for survival endpoints and hazard ratios (HR) with 95% confidence intervals were reported.

To reduce the bias due to the effects of confounding variables in the two cohorts, Propensity Score Matching (PSM) has been performed for DFS and OS. The patients have been matched 1:1 by age at diagnosis, nodal status, pT, and molecular subtype so 49 males have been matched with 49 women. DFS and OS have been calculated.

*p*-values < 0.05 were considered to be statistically significant.

Data were analyzed using SPSS software, version 25.0 for macOS (IBM Corp., Armonk, NY, USA).

## 3. Results

### 3.1. Study Population

The characteristics of the entire population, male and female, are presented in Table 1.

The mean age at diagnosis for male BC was 68.6 years (±10.1). The first symptom was, in most of the male BC cases, the presence of a unilateral breast lump (73.5% of the patients); less often nipple discharge or nipple retraction (26.5% of the patients).

Six males carried genetic mutations: four patients had a BRCA2 mutation, one patient a CHECK2 mutation, and one patient presented MLH1 mutation (associated with Lynch syndrome).

### 3.2. Tumor Characteristics

Males presented a smaller tumor size (mean 17.3 mm vs. 21.8 mm; *p* < 0.05) than women. In both populations, tumors were pT1 or pT2 in more than half of the cases. An amount of 14% of male BC were diagnosed at pT4 stage compared with only 6% of women (*p* = 0.01). In contrast, no men with pT3 tumors were present in our case series.

Males and females presented node-negative disease in 67.3% and 64.7%, respectively (*p* = 0.79). Axillary surgery was not performed in 25 women and in a man due to poor general conditions.

The most common histological features in male BC were the non-special type (NST) ductal carcinoma subtype with high nuclear grade (G3) and elevated Ki67. A lower rate of lobular tumors was noticed among male BC compared with females (4.1% vs. 10%, respectively; *p* < 0.05). Nearly 90% of male BC were IHC luminal A or B subtypes, and we had no cases of basal-like male BC (vs. 8.9% in female BC; *p* = 0.06). According to IHC classification, male BC was more frequently luminal B than FBC (53.1% vs. 24.5%, *p* = 0.04).

### 3.3. Surgery and Adjuvant Treatment

A relevant difference between male BC and FBC is the type of surgery performed. In fact if all men with BC underwent mastectomy, this type of surgery was performed in only one third of the women (*p* < 0.05). Axillary surgery was carried out following the indications given by national and international guidelines. Specifically, most of our male patients (*n* = 33) presented clinically N0 at diagnosis, and BLNS was performed, while ten patients had lymph node involvement, so they underwent ALND. In 5 cases, axillary dissection was performed following the finding of lymph node metastasis at BLNS in patients who underwent a mastectomy. In one case, axillary surgery was omitted due to the patient’s poor general clinical condition.

Most male BC patients received adjuvant endocrine therapy (AET) with tamoxifen (73.5%). Six patients received no AET due to personal refusal. In agreement with the initial selection criteria (post-menopausal patients), the majority of FBC patients had AET with aromatase inhibitors (*p* < 0.05). AET dropout rate due to side effects was 16.3% for males compared to 7.6% for women (*p* = 0.04).

Radiotherapy on the chest wall was indicated in 16.3% of male BC compared with 4.1% of FBC (*p* < 0.05), probably due to the higher rate of pT4 tumors diagnosed in males.

On the contrary, the adjuvant chemotherapy rate was similar in both male and female groups, and accounts for 40% in both groups.

### 3.4. Disease Recurrence and Overall Survival Analysis

The mean follow-up was 66 months (±30.9).

Univariate analysis for DFS performed on the entire female population identified the following risk factors for recurrence: nodal involvement, G3, tumoral diameter > 2 cm, Ki67 > 20%, and luminal B-type and basal-like IHC subtypes. The drop-out of AET had no apparent impact on DFS (Table 2).

Univariate analysis for DFS in male BC showed prognostically unfavorable factors tumor dimension > 20 mm, nodal involvement, G3, and luminal B IHC subtype. (Table 2).

**Figure 1:**
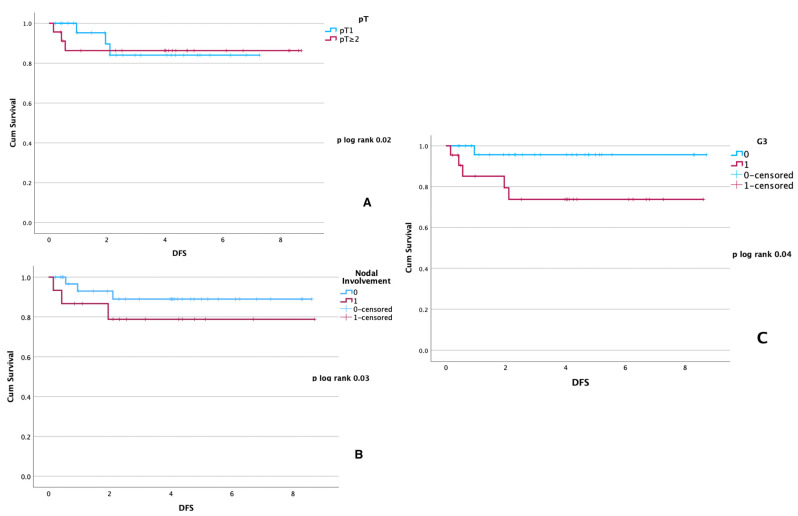
Male BC disease-free survival (DFS) expressed in years for: (**A**) tumor dimensions pT; (**B**) nodal involvement (0 = no nodal involvement, 1 = nodal involvement); (**C**) G3 (0 = G1 or G2; 1 = G3).

In male BC, multivariate Cox regression analysis for DFS confirmed tumoral dimensions > 20 mm, nodal involvement, and G3 as independent negative prognostic factors in male BC (Table 3) (Figure 1). In FBC, tumor dimension > 20 mm, and nodal involvement were statistically significant at multivariate analysis for DFS (Table 3).

OS data of FBC obtained from univariate analysis showed that pT3, histologic grade ≥ 2, and basal-like tumors resulted in reduced survival (Table 4). Focusing only on male BC, only histologic grade ≥ 2 was associated with worse OS at univariate analysis (Table 4). These results have not been confirmed at multivariate analysis in both FBC and male BC.

**Figure 2:**
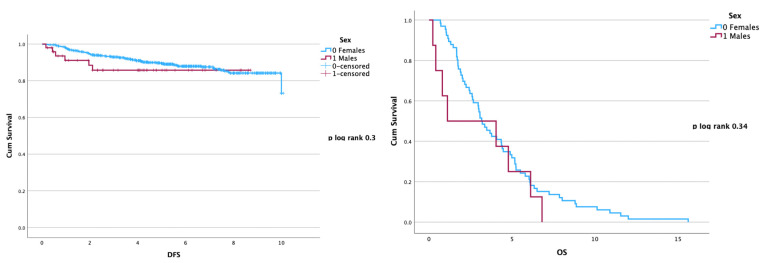
DFS and OS (both expressed in years) by sex: male vs. female in the entire population.

Comparing FBC and male BC, no significant differences have been identified in terms of DFS (*p* = 0.3) and OS (*p* = 0.34) (Figure 2), with a similar ten years-relapse rate (12% male BC vs. 12.4% female BC).

**Figure 3:**
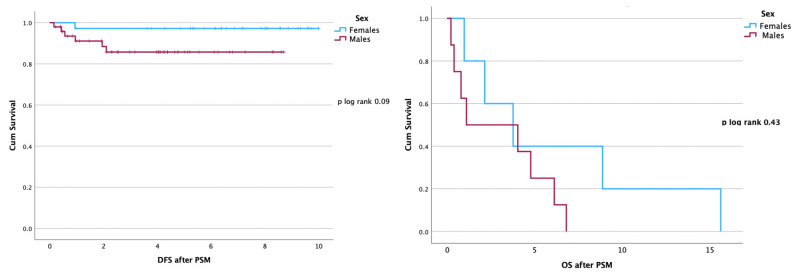
DFS and OS by sex after applying Propensity Score Matching. No significant differences were seen.

PSM by age at diagnosis, nodal status, pT, and molecular subtype had been performed to balance the two cohorts eliminating potential confounders; after matching, no differences in OS (*p* = 0.43) and in DFS (*p* = 0.09) were seen in the two groups (Figure 3).

## 4. Discussion

Male BC is a rare disease representing less than 1% of all cancer diagnoses worldwide and less than 1% of male tumors, even if the incidence is slightly rising [1,2,5,22,23]. In our study, male BC occurred at a mean age of 68.7 in accordance with the literature; male BC globally occurs at an older age than in women and the mean age at diagnosis is 67 years old [24,25].

Male BC shares major risk factors with FBC with the addition of all causes of hyperestrogenism [7].

Genetically, the BRCA 2 gene mutation is more prevalent in men with breast cancer, resulting in an increased risk of developing upper gastrointestinal cancer, prostate cancer, and BC later in life [6]. In our study, six male patients (4%) carried BRCA2 mutation, 1 CHECK2 mutation, and 1 MSH1 mutation. As McClurg et al. [26] reported in their recent review, genetic mutations, in particular BRCA1/2, are strongly related to development of breast cancer in males. Unfortunately, there are no clinical trials currently ongoing regarding BRCA-positive male breast cancer. Even when considering retrospective studies, the sample size is still low. In fact, the study with a higher sample size is the study conducted by Silvestri in which 44 men with BRCA1 mutation and 375 with BRCA2 mutation were included [27]; however, the purpose of the study was not to identify survival differences between men and women so survival analysis was not performed. In this context, larger sample sizes and prospective clinical trials are needed.

In our series, unilateral breast lump represented the first sign of male BC in 73.5% of cases, whereas in the female population, the diagnosis was mainly achieved by mammographic screening implemented in the last few years [6,7,10,14]. Regarding clinical features at diagnosis, some studies suggest that males usually present with a larger tumor diameter and more frequent nodal involvement than females [4,28]. On the contrary, in our patients, tumor dimension was significantly lower in male patients than in females. Nodal involvement showed a similar rate in both sexes. These results could be explained considering the increasing knowledge of BC reached thanks to several awareness campaigns made in the general population for the screening of FBC [28]. These strategies could have sensitized both female and male patients to come to the attention of physicians in case of a breast lump or other early symptoms. However, when considering tumor dimension, anatomopathological assessment demonstrated the absence of pT3 in our male patients in favor of a relatively higher percentage of pT4 than FBC. This finding can be explained by referring to the fact that the mammary gland in males is generally less represented than in females, so the presence of a tumor >5 cm more easily results in infiltration of the skin or the thoracic wall, effectively leading to a higher rate of pT4 diagnosis in males.

In our series of male BC, the least frequent histologic type is the lobular histotype accounting for 4.1% of all tumors (vs. 10% in females), and the most frequent IHC subtype is luminal, whereas basal-like and HER2+ BC are less represented (0% and 10%, respectively) in agreement with Johansson et al. and a SEER database analysis published in 2012 and other studies [9,29,30].

Regarding surgical treatment, all males underwent mastectomy compared with 36% of women. The twofold likelihood of males receiving mastectomy is probably due to the mammary gland tumor ratio and the higher frequency of pT4 tumors, thus making mastectomy the most appropriate surgery in accordance with guidelines [3,31].

In line with the literature, endocrine therapy plays a leading role among systemic adjuvant treatments in hormone-responsive tumors, in both males and females, reducing the rate of recurrence and increasing survival [32]. Recent studies based on female patients show how the prognostic benefits of AET appear to be further enhanced by the introduction of AET for 7–10 years [33,34].

In men, tamoxifen represents the most employed drug, followed by aromatase inhibitors associated or not with ovarian suppression function (OSF) with GnRH agonists [13,33].

Besides the evident benefits, AET is burdened with non-negligible side effects that may lead to drug discontinuation. As mentioned in a recent paper published by our group, the main side effects that can lead to drug drop-outs in up to 11.3% of patients taking AI and 13.8% of premenopausal patients taking tamoxifen ±OFS are related to anti-estrogen action such as hot flashes, vaginal dryness, sexual dysfunction, and dyspareunia, increased thrombotic risk, weight gain, arthralgias, and insomnia [35].

In the present study, AET was well tolerated by women with a dropout rate of less than 10%, probably due to the inclusion of only postmenopausal women. On the contrary, the adherence to AET was statistically lower for males with a discontinuation rate of 16% due to the side effects cited above, in particular decreased libido, weight gain, and hot flashes. Interestingly, such discontinuation had no impact on the risk of recurrence. This finding needs more data to be confirmed but could reflect tumor heterogeneity and complexity. In this regard, several trials are underway evaluating genomic differences between FBC and male BC, and testing the efficacy of drugs directed against the androgen receptor [36].

Forty percent of both women and men received adjuvant chemotherapy. The literature has not demonstrated a significant difference between men and women concerning the use of chemotherapy for BC. A recent study from Giordano et al. including 135 patients with a median follow-up of 14 years, pointed out that adjuvant chemotherapy (alone or associated with endocrine therapy) reduces male BC mortality by 43% and also significantly reduces the risk of recurrence [29].

Data on male BC survival outcomes widely vary in the literature and are often based on low-number studies; on the contrary, studies that include a higher number of patients date back many years when there were no screening programs and treatment options were different [37].

Taking into account recent data, it has been shown that the cumulative 5-year survival rate of male BC is between 36 and 66% and in some studies appears statistically lower than the survival rate in women (80–86%), mainly due to the more advanced stage at presentation of male BC [12,38]. In contrast, the study by Miao H et al. that includes 459,846 women and 2665 men from six population-based cancer registries from 1970 to 2007 noted that even if males presented cancer more frequently at a more advanced stage than females, the formers have better specific disease survival than the latter [39]. As Gucalp et al. pointed out, many studies claiming that male BC has a worse prognosis than FBC use OS as the survival outcome [12,40,41]. This parameter may not be very accurate for survival analysis as males are burdened with more comorbidities than females and have a shorter life expectancy [39]. Moreover, many studies included patients from SEER database were many prognostic factors were not systematically collected, as HER2 status, adjuvant treatments, and comorbidity [38,42].

In our study, based on a real-life cohort of female and male patients, the prognostic factors for worse DFS were equal for both sexes. In females, the main factors related to recurrence are tumoral diameter > 20 mm and nodal involvement; similarly, in males, the tumor characteristics significantly and independently correlated with worse DFS were tumor diameter > 20 mm, nodal involvement, and G3.

In our population, no significant differences in survival and risk of recurrence at ten years between males and females had been seen, with an almost similar 10-year relapse rate in the two groups, as well as in other studies [41,43,44].

To eliminate confounding factors and selection bias, we applied the PSM by pairing females with males. In contrast to Scomersi et al. who obtained a higher risk of recurrence in male BC after PSM, we observed no differences in DFS and OS in our case series between females and males [45]. This result is probably due to the selection criteria we used to choose female patients which made the two populations very similar at the start. Our study has some limitations as a retrospective study design and missing data regarding disease-specific survival.

To the best of our knowledge, ours is one of the few real-life studies in the literature with a discrete sample size of male BC and an average follow-up of more than 5 years. Furthermore, our patients were treated by the same surgical team and with the same medical and surgical treatment protocols. However, studies with a larger sample size are needed to confirm our data.

The future perspective of our study is to expand the case series of males and evaluate the impact of BRCA1/2 mutation on clinicopathological features and survival outcomes.

## 5. Conclusions

According to our preliminary data, we can assume that male BC and FBC have similar prognostic factors and survival outcomes. The drop-out rate of AET was higher in males and the side effects were the main reason for drug discontinuation; the real challenge will be to identify male BC characteristics and develop more specific and tolerable therapies.

Like FBC, male BC presents great complexity and heterogeneity. Further randomized prospective clinical trials are needed to clarify the biology and molecular pathways to achieve more targeted and tailored treatments.

## Figures and Tables

**Table 1:** Patients’ tumoral characteristics. FBC: female breast cancer; male BC male breast cancer; sd: standard deviation; NST: non-special type; AET: adjuvant endocrine therapy.

Variables	FBC (*n* = 680)	Male BC (*n* = 49)	*p*-Value
**Age at diagnosis** (years; SD)	66.6 (±11.2)	68.6 (±10.1)	0.25
**Type of surgery**			
Conservative	434 (63.8%)	0	**<0.05**
Mastectomy	246 (36.2%)	49 (100%)	**<0.05**
**Tumor diameter (mm; SD)**	21.5 (±12.1)	17.3 (±7.8)	**<0.05**
pT			
pT1	375 (54.3%)	26 (53.1%)	0.88
pT2	251 (36.4%)	16 (32.7%)	0.64
pT3	22 (3.2%)	0	0.39
pT4	32 (6.1%)	7 (14.2%)	**0.01**
**Nodal status (N)**			
pN0	440 (64.7%)	33 (67.3%)	0.72
pN+	215 (31.6%)	15 (30.6%)	0.79
pNx	25 (3.7%)	1 (2.1%)	0.89
**Tumor grade (G)**			
G1	71 (10.4%)	8 (16.3%)	0.22
G2	253 (37.2%)	19 (38.8%)	0.87
G3	356 (52.4%)	22 (44.9%)	0.31
**Ki67 > 20%**	372 (53.9%)	29 (59.1%)	0.3
**Histological type**			
NST	500 (72.5%)	36 (73.5%)	0.81
Lobular	69 (10%)	2 (4.1%)	**<0.05**
Other	121 (17.5%)	11 (22.4%)	0.23
**Molecular subtypes**			
Luminal A	300 (43.5%)	18 (36.7%)	0.07
Luminal B	169 (24.5%)	26 (53.1%)	**0.04**
Luminal B HER2+/HER2+	160 (23.1%)	5 (10.2%)	0.09
Basal-like	51 (8.9%)	0 (0%)	0.06
**AET**	592 (87%)	46 (93.9%)	
Tamoxifen	36 (5.3%)	36 (73.5%)	**<0.05**
Aromatase Inhibitors	556 (81.8%)	4 (8.2%)	**<0.05**
None	88 (12.9%)	6 (12.3%)	0.25
**AET drop-out rate**	48 (7.6%)	8 (16.3%)	**0.04**
**Adjuvant radiotherapy**			
Breast	434 (63.8%)	0	**<0.05**
Thoracic wall/nodes	28 (4.1%)	8 (16.3%)	**<0.05**
None	218 (32.1%)	41 (83.7%)	**<0.05**
**Adjuvant chemotherapy**	281 (40.7%)	20 (40.8%)	1

**Table 2:** Univariate analysis for DFS: (a) FBC, (b) male BC. HR: hazard ratio; CI: confidence interval.

	(a) Females			(b) Males		
Variables	HR	CI	*p* Value	HR	CI	*p* Value
**Tumor stage**						
pT1	1			1		
pT2	1.62	1.1–2.4	**0.03**	1.97	1.4–2.7	**<0.05**
pT3	2.02	0.81–4.98	0.13	-	-	-
pT4	4.95	2.78–8.82	**<0.05**	1.48	1.3–2.02	**0.04**
**Nodal involvement**	2.22	1.8–2.02	**0.03**	2.55	1.8–2.02	**0.02**
**Grade**						
G1	1			**1**		
G2	1.23	0.84–2.56	0.73	1.32	0.73–3.45	0.34
G3	2.71	1.71–4.34	**<0.05**	1.84	1.2–3.6	**0.04**
**Ki67 value**						
Ki67 < 20%	1			**1**		
Ki67 > 20%	2.02	1.28–3.17	**<0.05**	4.24	0.49–36.3	0.19
**Molecular subtypes**						
Luminal A	1			1		
Luminal B	2.55	1.69–3.85	**<0.05**	2.47	1.07–5.73	**0.03**
Luminal B HER2+/HER2+	0.78	0.47–1.28	0.32	1.49	0.49–4.54	0.47
Basal-like	2.54	1.38–4.67	**<0.05**	**-**	**-**	**-**
**AET drop-out**	1.29	0.65–2.58	0.47	2.45	0.45–13.3	0.29

**Table 3:** Multivariate analysis Cox regression for DFS: (a) FBC, (b) male BC. HR: hazard ratio; CI: confidence interval.

	(a) Females			(b) Males		
Variables	HR	CI	*p* Value	HR	CI	*p* Value
pT ≥ 2	1.54	1.24–1.91	**<0.05**	1.50	1.15–1.94	**0.02**
Nodal involvement	6.66	3.98–11.15	**<0.05**	6.43	3.55–11.67	**<0.05**
G3	1.67	0.97–2.71	0.06	1.94	1.08–3.51	**0.01**
Luminal B	1.15	0.76–1.29	0.73	1.22	0.66–1.21	0.4
Basal-like	1.74	0.84–3.78	0.07	-	-	-

**Table 4:** Univariate analysis for OS: (a) FBC, (b) male BC. HR: hazard ratio; CI: confidence interval; AET: adjuvant endocrine therapy.

	(a) Females			(b) Males		
Variables	HR	CI	*p* Value	HR	CI	*p* Value
**Tumor stage**						
pT1	1			1		
pT2	1.40	0.80–2.43	0.23	1.12	0.88–1.66	0.24
pT3	2.5	1.06–5.89	**0.03**	- *	-	**-**
pT4	1.02	0.79–1.31	0.89	1.01	0.79–1.30	0.90
**Nodal involvement**	1.32	0.82–2.11	0.25	1.65	0.34–8.34	0.55
**Grade**						
G1	1			1		
G2	4.09	1.32–12.6	**0.02**	3.94	1.29–12.2	**0.02**
G3	2.88	1.11–7.47	**0.03**	2.93	1.13–7.57	**0.03**
**Ki67 value**						
Ki67 < 20%	ref			1		
Ki67 > 20%	1.22	0.74–1.98	0.44	4.14	0.73–23.5	0.11
**Molecular subtypes**						
Luminal A	1			1		
Luminal B	1.24	0.69–2.19	0.46	1.27	0.79–2.02	0.31
Luminal B HER2+/HER2+	0.93	0.67–1.27	0.69	0.93	0.68–1.28	0.67
Basal-like	1.42	1.06–1.90	**<0.05**	- *	-	**-**
**AET drop-out**	1.02	0.24–4.2	0.97	0.32	0.03–2.71	0.29

* In our case series of male BC, no pT3 and basal-like tumors were diagnosed.

## Data Availability

The data presented in this study are available on request from the corresponding author on reasonable request. The data are not publicly available due to privacy policies.

## References

[1] Mangone L., Ferrari F., Mancuso P., Carrozzi G., Michiara M., Falcini F., Piffer S., Filiberti R.A., Caldarella A., Vitale F. (2020). Epidemiology and biological characteristics of male breast cancer in Italy. Breast Cancer.

[2] Humphries M.P., Rajan S.S., Honarpisheh H., Cserni G., Dent J., Fulford L., Jordan L.B., Jones J.L., Kanthan R., Litwiniuk M. (2017). Characterisation of male breast cancer: A descriptive biomarker study from a large patient series. Sci. Rep..

[3] Sousa B., Moser E., Cardoso F. (2013). An update on male breast cancer and future directions for research and treatment. Eur. J. Pharmacol..

[4] Korde L.A., Zujewski J.A., Kamin L., Giordano S., Domchek S., Anderson W.F., Bartlett J.M., Gelmon K., Nahleh Z., Bergh J. (2010). Multidisciplinary Meeting on Male Breast Cancer: Summary and Research Recommendations. J. Clin. Oncol..

[5] Losurdo A., Rota S., Gullo G., Masci G., Torrisi R., Bottai G., Zuradelli M., Gatzemeier W., Santoro A. (2017). Controversies in clinicopathological characteristics and treatment strategies of male breast cancer: A review of the literature. Crit. Rev. Oncol..

[6] Silvestri V., Barrowdale D., Mulligan A.M., Neuhausen S.L., Fox S., Karlan B.Y., Mitchell G., James P., Thull D.L., Zorn K.K. (2016). Male breast cancer in BRCA1 and BRCA2 mutation carriers: Pathology data from the Consortium of Investigators of Modifiers of BRCA1/2. Breast Cancer Res..

[7] Sanguinetti A., Polistena A., Lucchini R., Monacelli M., Galasse S., Avenia S., Triola R., Bugiantella W., Cirocchi R., Rondelli F. (2016). Male breast cancer, clinical presentation, diagnosis and treatment: Twenty years of experience in our Breast Unit. Int. J. Surg. Case Rep..

[8] Saita C., Yamaguchi T., Horiguchi S.-I., Yamada R., Takao M., Iijima T., Wakaume R., Aruga T., Tabata T., Koizumi K. (2018). Tumor development in Japanese patients with Lynch syndrome. PLoS ONE.

[9] Greif J.M., Pezzi C.M., Klimberg V.S., Bailey L., Zuraek M. (2012). Gender Differences in Breast Cancer: Analysis of 13,000 Breast Cancers in Men from the National Cancer Data Base. Ann. Surg. Oncol..

[10] Uslukaya O., Gumus M., Gumus H., Bozdag Z., Turkoglu A. (2016). The Management and Outcomes of Male Breast Cancer. J. Breast Heal..

[11] Chen X., Liu X., Zhang L., Li S., Shi Y., Tong Z. (2013). Poorer Survival of Male Breast Cancer Compared with Female Breast Cancer Patients May Be Due to Biological Differences. Jpn. J. Clin. Oncol..

[12] National Cancer Institute Surveillance, Epidemiology and End Results Program. https://seer.cancer.gov/statfacts/html/breast.html.

[13] Leon-Ferre R.A., Giridhar K.V., Hieken T.J., Mutter R.W., Couch F.J., Jimenez R.E., Hawse J.R., Boughey J.C., Ruddy K.J. (2018). A contemporary review of male breast cancer: Current evidence and unanswered questions. Cancer Metastasis Rev..

[14] Hammond M.E.H., Hayes D.F., Dowsett M., Allred D.C., Hagerty K.L., Badve S., Fitzgibbons P.L., Francis G., Goldstein N.S., Hayes M. (2010). American Society of Clinical Oncology/College of American Pathologists Guideline Recommendations for Immunohistochemical Testing of Estrogen and Progesterone Receptors in Breast Cancer. J. Clin. Oncol..

[15] Wolff A.C., Hammond M.E.H., Schwartz J.N., Hagerty K.L., Allred D.C., Cote R.J., Dowsett M., Fitzgibbons P.L., Hanna W.M., Langer A. (2007). American Society of Clinical Oncology/College of American Pathologists Guideline Recommendations for Human Epidermal Growth Factor Receptor 2 Testing in Breast Cancer. J. Clin. Oncol..

[16] NCCN Guidelines Version 4.2023 Breast Cancer. www.nccn.org.

[17] Nguyen P.L., Taghian A.G., Katz M.S., Niemierko A., Raad R.F.A., Boon W.L., Bellon J.R., Wong J.S., Smith B.L., Harris J.R. (2008). Breast Cancer Subtype Approximated by Estrogen Receptor, Progesterone Receptor, and HER-2 Is Associated with Local and Distant Recurrence After Breast-Conserving Therapy. J. Clin. Oncol..

[18] Goldhirsch A., Winer E.P., Coates A.S., Gelber R.D., Piccart-Gebhart M., Thürlimann B., Senn H.-J. (2013). Personalizing the treatment of women with early breast cancer: Highlights of the St Gallen International Expert Consensus on the Primary Therapy of Early Breast Cancer 2013. Ann. Oncol..

[19] Gnant M., Harbeck N., Thomssen C. (2011). St. Gallen 2011: Summary of the Consensus Discussion. Breast Care.

[20] Harbeck N., Thomssen C., Gnant M. (2013). St. Gallen 2013: Brief Preliminary Summary of the Consensus Discussion. Breast Care.

[21] NCCN Breast Cancer Guidelines 2015. www.nccn.org.

[22] Masci G., Caruso M., Caruso F., Salvini P., Carnaghi C., Giordano L., Miserocchi V., Losurdo A., Zuradelli M., Torrisi R. (2015). Clinicopathological and Immunohistochemical Characteristics in Male Breast Cancer: A Retrospective Case Series. Oncol..

[23] Humphries M.P., Jordan V.C., Speirs V. (2015). Obesity and male breast cancer: Provocative parallels?. BMC Med..

[24] Hill T.D., Khamis H.J., Tyczynski J.E., Berkel H. (2005). Comparison of Male and Female Breast Cancer Incidence Trends, Tumor Characteristics, and Survival. Ann. Epidemiol..

[25] Liu D., Xie G., Chen M. (2013). Clinicopathologic characteristics and survival of male breast cancer. Int. J. Clin. Oncol..

[26] Patten D.K., Sharifi L.K., Fazel M. (2013). New Approaches in the Management of Male Breast Cancer. Clin. Breast Cancer.

[27] McClurg D.P., Urquhart G., McGoldrick T., Chatterji S., Miedzybrodzka Z., Speirs V., Elsberger B. (2022). Analysis of the Clinical Advancements for *BRCA*-Related Malignancies Highlights the Lack of Treatment Evidence for *BRCA*-Positive Male Breast Cancer. Cancers.

[28] Cutuli B., Le-Nir C.C.-S., Serin D., Kirova Y., Gaci Z., Lemanski C., De Lafontan B., Zoubir M., Maingon P., Mignotte H. (2010). Male breast cancer. Evolution of treatment and prognostic factors. Analysis of 489 cases. Crit. Rev. Oncol..

[29] 30th European Guidelines on Breast Cancer Screening and Diagnosis. https://healthcare-quality.jrc.ec.europa.eu/ecibc/european-breast-cancer-guidelines.

[30] Giordano S.H., Perkins G.H., Broglio K., Garcia S.G., Middleton L.P., Buzdar A.U., Hortobagyi G.N. (2005). Adjuvant systemic therapy for male breast carcinoma. Cancer.

[31] Johansson I., Killander F., Linderholm B., Hedenfalk I. (2014). Molecular profiling of male breast cancer—Lost in translation?. Int. J. Biochem. Cell Biol..

[32] Fentiman I.S. (2018). Surgical options for male breast cancer. Breast Cancer Res. Treat..

[33] Rossi L., McCartney A., De Santo I., Risi E., Moretti E., Malorni L., Biganzoli L., Di Leo A. (2019). The optimal duration of adjuvant endocrine therapy in early luminal breast cancer: A concise review. Cancer Treat. Rev..

[34] Villasco A., Accomasso F., D’alonzo M., Agnelli F., Sismondi P., Biglia N. (2021). Evaluation of the ability of the Clinical Treatment Score at 5 years (CTS5) compared to other risk stratification methods to predict the response to an extended endocrine therapy in breast cancer patients. Breast Cancer.

[35] Curigliano G., Burstein H.J., Winer E.P., Gnant M., Dubsky P., Loibl S., Colleoni M., Regan M.M., Piccart-Gebhart M., Senn H.-J. (2017). De-escalating and escalating treatments for early-stage breast cancer: The St. Gallen International Expert Consensus Conference on the Primary Therapy of Early Breast Cancer 2017. Ann. Oncol. Off. J. Eur. Soc. Med. Oncol..

[36] Rosso R., D’alonzo M., Bounous V.E., Actis S., Cipullo I., Salerno E., Biglia N. (2023). Adherence to Adjuvant Endocrine Therapy in Breast Cancer Patients. Curr. Oncol..

[37] Severson T.M., Zwart W. (2017). A review of estrogen receptor/androgen receptor genomics in male breast cancer. Endocr.-Relat. Cancer.

[38] Gucalp A., Traina T.A., Eisner J.R., Parker J.S., Selitsky S.R., Park B.H., Elias A.D., Baskin-Bey E.S., Cardoso F. (2019). Male breast cancer: A disease distinct from female breast cancer. Breast Cancer Res. Treat..

[39] Liu N., Johnson K.J., Ma C.X. (2018). Male Breast Cancer: An Updated Surveillance, Epidemiology, and End Results Data Analysis. Clin. Breast Cancer.

[40] Miao H., Verkooijen H.M., Chia K.-S., Bouchardy C., Pukkala E., Larønningen S., Mellemkjær L., Czene K., Hartman M. (2011). Incidence and Outcome of Male Breast Cancer: An International Population-Based Study. J. Clin. Oncol..

[41] El-Tamer M.B., Komenaka I.K., Troxel A., Li H., Joseph K.-A., Ditkoff B.-A., Schnabel F.R., Kinne D.W. (2004). Men with Breast Cancer Have Better Disease-Specific Survival Than Women. Arch. Surg..

[42] Marchal F., Salou M., Marchal C., Lesur A., Desandes E. (2009). Men with Breast Cancer Have Same Disease-Specific and Event-Free Survival as Women. Ann. Surg. Oncol..

[43] Yao N., Shi W., Liu T., Siyin S.T., Wang W., Duan N., Xu G., Qu J. (2022). Clinicopathologic characteristics and prognosis for male breast cancer compared to female breast cancer. Sci. Rep..

[44] Foerster R., Foerster F.G., Wulff V., Schubotz B., Baaske D., Wolfgarten M., Kuhn W.C., Rudlowski C. (2011). Matched-pair analysis of patients with female and male breast cancer: A comparative analysis. BMC Cancer.

[45] Scomersi S., Giudici F., Cacciatore G., Losurdo P., Fracon S., Cortinovis S., Ceccherini R., Zanconati F., Tonutti M., Bortul M. (2021). Comparison between male and female breast cancer survival using propensity score matching analysis. Sci. Rep..

